# Characterization of PF-6142, a Novel, Non-Catecholamine Dopamine Receptor D1 Agonist, in Murine and Nonhuman Primate Models of Dopaminergic Activation

**DOI:** 10.3389/fphar.2020.01005

**Published:** 2020-07-07

**Authors:** Rouba Kozak, Tamás Kiss, Keith Dlugolenski, David E. Johnson, Roxanne R. Gorczyca, Kyle Kuszpit, Brian D. Harvey, Polina Stolyar, Stacey J. Sukoff Rizzo, William E. Hoffmann, Dmitri Volfson, Mihaly Hajós, Jennifer E. Davoren, Amanda L. Abbott, Graham V. Williams, Stacy A. Castner, David L. Gray

**Affiliations:** ^1^Global Research and Development, Pfizer Inc., Groton, CT, United States; ^2^Department of Comparative Medicine, Yale School of Medicine, New Haven, CT, United States

**Keywords:** prefrontal cortex, schizophrenia, working memory, pro-cognitive therapeutics, Parkinson’s disease

## Abstract

Selective activation of dopamine D1 receptors remains a promising pro-cognitive therapeutic strategy awaiting robust clinical investigation. PF-6142 is a key example from a recently disclosed novel series of non-catechol agonists and partial agonists of the dopamine D1/5 receptors (D1R) that exhibit pharmacokinetic (PK) properties suitable for oral delivery. Given their reported potential for functionally biased signaling compared to known catechol-based selective agonists, and the promising rodent PK profile of PF-6142, we utilized relevant *in vivo* assays in male rodents and male and female non-human primates (NHP) to evaluate the pharmacology of this new series. Studies in rodents showed that PF-6142 increased locomotor activity and prefrontal cortex acetylcholine release, increased time spent in wakefulness, and desynchronized the EEG, like known D1R agonists. D1R selectivity of PF-6142 was supported by lack of effect in D1R knock-out mice and blocked response in the presence of the D1R antagonist SCH-23390. Further, PF-6142 improved performance in rodent models of NMDA receptor antagonist-induced cognitive dysfunction, such as MK-801-disrupted paired-pulse facilitation, and ketamine-disrupted working memory performance in the radial arm maze. Similarly, PF-6142 reversed ketamine-induced deficits in NHP performing the spatial delayed recognition task. Of importance, PF-6142 did not alter the efficacy of risperidone in assays predictive of antipsychotic-like effect in rodents including pre-pulse inhibition and conditioned avoidance responding. These data support the continued development of non-catechol based D1R agonists for the treatment of cognitive impairment associated with brain disorders including schizophrenia.

## Introduction

D1 receptors (D1Rs) play a central role in important domains of cognitive function including spatial learning and memory, reversal, extinction, and incentive learning (Huang and Kandel, 1995; Rascol et al., 1999; Goldman-Rakic et al., 2000; Seamans et al., 2001; Williams and Castner, 2006; Takahashi et al., 2012; Wass et al., 2013) and D1R expression or signaling are compromised in a variety of psychiatric, neurological, and endocrine disorders including schizophrenia, drug addiction, and Parkinson’s disease (Haney et al., 1998; Wang et al., 1998; Mailman et al., 2001; Rosell et al., 2015; Papapetropoulos et al., 2018). Studies conducted by Sawaguchi and Goldman-Rakic using both agonists and antagonists (Sawaguchi et al., 1990; Sawaguchi and Goldman-Rakic, 1994) indicated that the modulation of working memory processes by mesocortical DA in primates is primarily mediated by D1Rs. Local administration of D1R antagonists into the dorsolateral prefrontal cortex (PFC) induced deficits in a working memory task whereas blockade of D2-like receptors gave no impairment. Subsequent studies revealed that a primary function of D1R activation is to enhance and stabilize task-related activity of PFC neurons (Williams and Goldman-Rakic, 1995).

Importantly, acute treatment with D1R agonists was shown to ameliorate age-related impairments of working memory (Arnsten et al., 1994) and to restore working memory performance in states characterized by prefrontal hypodopaminergia such as chronic stress, chronic neuroleptic treatment, and following low-dose 1-metil-4-fenil-1,2,3,6-tetrahidropiridin treatment (Schneider et al., 1994; Castner et al., 2000). D1R agonist therapy may ameliorate cognitive impairment by enhancing insufficient DA tone in the PFC of patients with schizophrenia (Abi-Dargham and Moore, 2003; Goldman-Rakic et al., 2004; Williams and Castner, 2006; Granado et al., 2008).

Although collective data suggests that increased signaling at D1R may benefit cognitive function in settings with dopaminergic deficits, there is experimental evidence showing that prefrontal dopamine (DA) transmission operates within a defined working range for efficient cortical function (Goldman-Rakic et al., 2000; Seamans and Yang, 2004; Williams and Castner, 2006; Cools and D’Esposito, 2011). On one hand, hyperactivation of D1Rs in the PFC of rodents induces impaired working memory (Zahrt et al., 1997), while on the other, a series of studies showed D1R agonist-produced divergent effects on cognitive performance. These observations led to the hypothesis of an inverted U-shaped dose response curve of D1R function in working memory (Vijayraghavan et al., 2007), an idea that is further supported by clinical studies (Mattay et al., 2003).

Despite the importance of this target, there has been a notable paucity of agents available clinically (Zhang et al., 2009). To date, only a few D1R selective agonists, such as dihydrexidine and ABT-431, have been approved for clinical use. In a set of small clinical studies these compounds yielded ambiguous results such as unchanged (Girgis et al., 2016) and enhanced (Rosell et al., 2015) working memory performance, as well as unchanged (Girgis et al., 2016) and increased (Mu et al., 2007) prefrontal perfusion in schizophrenia patients, possibly due to their PK and tolerability limitations.

Recent reports describe a new series of structurally novel compounds which selectively activate D1 and D5 receptors and have favorable PK (Davoren et al., 2018; Gray et al., 2018). Compounds from this series have entered clinical study where their favorable PK was confirmed, and they demonstrated efficacy in reducing motor symptoms of Parkinson’s disease (Papapetropoulos et al., 2018; Sohur et al., 2018) in single and repeated dose regimens and affects core aspects of cost-benefit decision making in humans (Soutschek et al., 2020a; Soutschek et al., 2020b). Herein, we characterize another exemplar of this new series of D1R-selective non-catechol agonists, PF-6142 (Davoren et al., 2018; Gray et al., 2018; Young et al., 2020) in preclinical assays evaluating its *in vivo* activity and further characterizing its pharmacological properties. This characterization includes studies which enable pharmacological comparison of PF-6142 to known D1R agonists in addition to behavioral and imaging paradigms which have not been previously explored with D1R agonists as summarized in **Table 1**.

**Table 1:** Summary of experimental paradigms and doses of PF-6142 used.

Experiment	Acute dose (mg/kg)	Subchronic dose (mg/kg)	Figure
ACh level, rat	10, SC	10, SC for 5 d	1
ACh level, mouse	10, SC	10, SC for 5 d	1
LMA, mouse	0.32, 1, 3.2, 10, SC	1.78, 3.2, 10, SC	2
qEEG/PSG, rat	1.0, 5.6, SC		3
PPI, mouse	1.78, SC		4
CAR, rat	1.78, SC		4
RAM, rat	0.01, 0.056, 0.178, 0.56, SC		5
SDR, NHP	0.0015, 0.015, 0.15, SC		5
PPF, rat	0.1, 0.3, 1.0, 3.0, IV, cumulative		6

ACh, acetylcholine; FDG-PET, fluorodeoxyglucose positron emission tomography; IV, intravenous; NHP, nonhuman primate; PSG, polysomnography; LMA, locomotor activity; PPI, prepulse inhibition; CAR, conditioned avoidance response; PPF, paired-pulse facilitation; RAM, radial arm maze; SC, subcutaneous; SDR, spatial delayed response task.

## Materials and Methods

### Drug Preparation Procedure

PF-6142 (synthesized in house by Pfizer Medicinal Chemistry group, Groton, CT; free base; Gray et al. (2018)) was dissolved in 5% dimethyl sulfoxide + 5% Cremophor EL + 90% sterile water or sterile saline + 0 to 3 molar equivalents of hydrochloric acid to a pH ~3–4 for subcutaneous administration both in rats and non-human primates (NHP). For intravenous (i.v.) administration, PF-6142 was dissolved in 20% (w/v) 2-hydroxypropyl-beta-cyclodextrin in sterile water for i.v. administration. Ketaset (Ford Dodge Animal Health, Iowa, USA; ketamine hydrochloride) was diluted in sterile saline. MK-801 (hydrogen maleate, Tocris Biosciences, Bristol, UK), A-77636 (hydrochloride, Tocris), and R (+)-SCH-23390 (hydrochloride, Sigma-Aldrich, MO, USA) were corrected for the weight of the salt and dissolved in sterile water or sterile saline. Risperidone (Sigma-Aldrich) was dissolved in 1% glacial acetic acid in saline for mouse pre-pulse inhibition (PPI) studies, or in 0.3% (w/v) tartaric acid in sterile saline for conditioned avoidance responding experiments (CAR) in rats. Both risperidone solutions were adjusted to pH 4 with sodium hydroxide. Dose volumes for rats were 1 ml/kg, except for the CAR study which was 2 ml/kg. Dose volumes for mice were 10 ml/kg. Urethane was administered at 1.5 mg/kg dissolved in sterile water. PF-6142 was dissolved in 12% (w/v) sulfobutylether-beta-cyclodextrin for oral (p.o.) dosing for the polysomnography study. LY-451-646 was dissolved in 10% Cremophor EL in sterile water.

### Animal Care

All animal procedures were approved by the Institutional Animal Care and Use Committee at Pfizer Inc. and conducted in accordance with the NIH Guide for the Care and Use of Laboratory Animals. All animals were at minimum 8 weeks of age at testing and were purchased from commercial vendors as follows: male C57BL/6J mice, male DRD1a wild-type (WT) and D1 DA receptor knockout mice (DRD1a-905781) from The Jackson Laboratory (Bar Harbor, ME); male CD-1 mice, male Fisher-344 rats and male Long-Evans rats from Charles River Laboratories (Kingston, NY); male Sprague-Dawley rats from Harlan Laboratories (Indianapolis, IN) for electroencephalography and polysomnography, as well as paired-pulse facilitation (PPF) studies and from Charles River Laboratories (Kingston, NY) for microdialysis studies. Rodents were group-housed in environmentally controlled animal quarters (light/dark-6:00 am/6:00 pm) and were acclimated to the facility prior to testing. Access to food and water was provided ad libitum to all rodents, except for the food restricted rats used for the radial arm maze (RAM) study and PET imaging.

An adult aged cohort of 10 male and female rhesus monkeys (Mucaca Mullata) were used for the spatial delayed response (SDR) task. They were maintained in accordance with the Yale/Animal Care and Use Committees and federal guidelines for the care and use of nonhuman primates and were fed their full allotment of standard monkey diet (Harlan Teklad Monkey Diet, Madison, WI, USA) and fruit/vegetables prior to, during, and following the experiment described herein. Animals received their normal allotment of biscuits immediately following cognitive testing and were given species appropriate environmental enrichment such as foraging devices and safe items to play with.

### Experimental Design and Statistical Analysis

#### Prefrontal Cortex Acetylcholine (aCh) Levels Determined *via* Microdialysis in Rat and Mouse

##### Surgery

Adult male Sprague-Dawley rats (280–360 g) were obtained from Charles-River Laboratories, Raleigh, NC and male C57BL/6J DRD1a WT and D1 DA receptor knockout mice (DRD1a-905781) were obtained from The Jackson Laboratories (Bar Harbor, ME). Animals were housed on a 12-h light/dark cycle with free access to food and water and allowed to acclimate for at least 5 d after arrival. Aseptic technique was used during the surgical procedure in order to prevent infection. On the day of the procedure animals were anesthetized with isoflurane (4%) and their heads shaved. The animals were then placed into a Kopf stereotaxic frame, the surgical area disinfected by swabbing with Provodine solution, and the area isolated with a sterile surgical drape. Anesthesia was maintained with isoflurane (2.5%–3%) delivered through a nose cone using a Univentor 400 anesthesia unit. Animals were given a 0.1 ml subcutaneous (s.c.) injection of Metacam (NSAID, meloxicam, 5 mg/ml, Boehringer Ingelheim) as a post-operative analgesic. Marcaine (bupivacaine, 0.5%, Hospira, Lake Forest, IL), a long acting local anesthetic, was administered s.c. at the surgical area to minimize pain and discomfort.

A 1.5–2 cm incision was made along the midline of the skull, beginning from a point just behind the eyes and running posterior. The skin was retracted with hemostats and the skull was further exposed using blunt dissection with cotton swabs. Bleeding capillaries were cauterized, and the skull dried with a sterile gauze sponge.

A microdialysis guide cannula [Bioanalytical Systems Inc (BAS), West Lafayette, IN, part # MD-2251] was placed into a guide holder on the stereotaxic frame and positioned over “Bregma”. The guide cannula was positioned over the PFC (A-P, +3.2 mm; M-L, +0.7 mm, left, relative to bregma) and the location marked on the skull. Using a 0.7 mm burr, a hole was made in the skull at the cannula position. To facilitate attachment with dental cement, an additional three holes were made surrounding the cannula hole to accept bone screws. The three self-tapping bone screws were inserted, and the cannula positioned over the cannula hole then slowly lowered to a depth −1.3 mm below the surface of the dura. The guide cannula was then fixed to the skull using acrylic dental cement.

Microdialysis probes were inserted one to 2 d after guide implantation. Prior to insertion, BAS probes (part # MD2204, 4 mm) were flushed at 2 ul/min for approximately 15 min with artificial CSF (aCSF) of the following composition: 147 mM NaCL, 1.3 mM CaCl2, 2.7 mM KCl, and 1 mM MgCl_2_. Animals were lightly anesthetized with isoflurane and the probe inserted. One to 2 h after insertion the probe flow was reduced to 0.3 µl/min and the animals allowed to recover overnight. At approximately 7:30 AM on the day after probe insertion the flow of aCSF through the probe was increased to 2 µl/min. Note- that in studies where HPLC-EC was used for **aCh** analysis, 100 nM neostigmine was added to the perfusion solution. After a stabilization period (typically around 1.5–2 h) several baseline samples were collected (15–30-min intervals) to establish an “average” basal level after which drug treatment was initiated. Samples were either collected on-line for analysis of ACh content by high performance liquid chromatography (HPLC) in conjunction with electrochemical detection (EC) or collected off-line for simultaneous determination of ACh content by liquid chromatography tandem mass spectrometry (LC-MS/MS).

##### Sample Analysis

###### HPLC/EC

For conventional analysis, ACh was analyzed by high performance liquid chromatography (HPLC) utilizing a modification of the BAS ACh-choline assay kit (BAS part # MF-8910). Note that for this analysis procedure 100 nM neostigmine bromide was added to the probe perfusion solution to increase the detection reliability of ACh. Briefly, ACh was separated at a flow rate of 1 ml/min and a temperature of 28°C on two 10 cm ACh analytical columns (BAS part # MF-6150) connected in series, using a mobile phase containing 35 mM Na_2_HPO_4_, 0.1 mM EDTA, and 0.005% ProClin^®^ and adjusted to pH 8.5 with phosphoric acid. ACh was then converted in a post-column acetylcholinesterase-choline oxidase immobilized enzyme reactor (BAS part # MF-6151) to hydrogen peroxide, which was detected electrochemically at a platinum electrode maintained at a potential of +0.5 V vs. Ag/AgCl. Chromatography data were collected and quantified by comparison to known standard concentrations using EZChrom Elite software (Agilent Technologies, Inc, Santa Clara, CA). Chromatography data for individual samples is archived on the Pfizer server \\groamrapp285\ezchrom.

###### LC-MS/MS

Samples were collected off-line and dialysate ACh and histamine levels were determined using LC)-MS/MS and in the absence of locally perfused neostigmine. Microdialysates (30 µl sample volume) were collected at 15-min intervals into glass vials containing 4 µl of 10% acetic acid using a refrigerated fraction collector then stored frozen at −80°C for later analysis. Prior to analysis, deuterated Acetylcholine-1,1,2,2-d4 bromide (200 ng/ml) and deuterated Histamine-α, α, β, β-d4 dihydrochloride (1,000 ng/ml) were added to each sample as an internal standard in a volume of 70 µl. Analytes (10 µl injected sample volume) were separated on a Waters Atlantic Hilic column (100 x 2.1 mm, 3 µm particle size) at a temperature of 25 °C using a Waters Acquity Ultraperfomance liquid chromatograph (Waters Corporation, Milford, MA). Separation was achieved at a flow rate of 0.22 ml/min using a binary solvent gradient elution where solvent A consisted of 20 mM ammonium formate in 1% formic acid, pH 3.4 and solvent B was 100% acetonitrile. Each cycle began with a linear gradient running from 10% to 70% solvent A over 3 min and was then held at 70% solvent A for 1.5 min before returning to 10% solvent A in 0.5 min. The effluent from the LC column was directed at the electrospray interface of the mass spectrometer. LC-MS/MS analyses were performed using a Sciex API 3000 triple quadrapole mass spectrometer equipped with a turboionspray source (AB Sciex, Framingham, MA). The ion spray voltage was set at 1,500 V and the source temperature at 450° C. The mass spectrometer was operated in the positive ion electrospray mode with the following parameters: declustering potential, 25 V; focusing potential, 100 V; entrance potential, 5 V; collision cell exit potential, 22 V. Nitrogen was used for both the curtain and collision gas with an ion energy of 6 and 8 eV, respectively. ACh, d4 ACh, histamine, and d4 histamine were monitored using multiple reaction monitoring (MRM) mode. The MRM transitions m/z 146.2→87.1 and 150.2→91.3 were sequentially monitored for the detection of ACh and deuterated ACh, respectively. The MRM transitions m/z 112.2→95.1 and 116.1→99.0 were sequentially monitored for the detection of histamine and deuterated histamine, respectively, LC-MS/MS data were collected and analyzed by comparison to known standard concentrations using Analyst software version 1.4.1. (AB Sciex, Framingham, MA). Added details of the LC-MS/MS procedure are in E-Notebook VBN#00702189 in the Published PDF/Root/Research/Groton/E-H/Gorczyca, Roxanne R VBN#00702189/Methods/LCMS-MS protocol ACh, and HA microdialysate/PDFs/20120105-1412-v2-LCMS-MS protocol ACh and HA microdialysate.

##### Data Analysis

Statistical analyses were performed using Graph Pad Prism 5 software. Raw time course data was normalized for variation in basal levels among animals by converting each time point to a ratio of the response over the average baseline level (3–5 samples prior to 1st treatment) for each animal. It is referred to as fraction of baseline.

##### Statistical Tests

To test for significant changes from baseline, three fraction of baseline values were averaged for each treatment period. Changes from basal were evaluated using repeated measures one-way ANOVA with Dunnett’s post-hoc tests. To test for significant differences in time course data a repeated measures two-way ANOVA with Bonferroni correction for multiple testing were performed.

#### Mouse Locomotor Activity (LMA)

Locomotor activity data were measured by an automated infrared photo-beam system in sound attenuating chambers controlled by Versamax^®^ software, provided by Accuscan Instruments Inc. (Columbus, Ohio), which quantified beam breaks similar to the methods used previously (Xu et al., 2000). To test D1 receptor selectivity in a pharmacologic model, C57BL/6J mice were habituated to the apparatus for 90 min, followed by pretreatment with vehicle or SCH-23390 (0.01, 0.032, 0.1 0.32, s.c.) and returned to the apparatus for 30 min. After the 30-min pretreatment period mice were administered vehicle (s.c.) or PF-6142 (0.32, 1, 3.2, 10 mg/kg, s.c.) and returned to the apparatus at which time activity was measured for a 2-h period (**Figure 2A**). Data were compared against the vehicle + PF-6142 (10 mg/kg) group using a repeated measures one-way ANOVA with a Dunnett’s post-test. The repeated treatment data were obtained from C57BL/6J mice that were habituated to the apparatus for 90 min, dosed with vehicle or PF-1642 (1.78, 3.2, 10 mg/kg, s.c.), returned to the chamber and measured for activity for 2 h. The same mice were treated for five consecutive days and data are presented in **Figure 2B**. Data were compared against the vehicle group using a repeated measures one-way ANOVA with a Dunnett’s post-test. To test D1 receptor selectivity in a genetic model, DRD1a WT and knockout mice (D1 KO) were habituated to the apparatus for 90 min, pretreated with vehicle or SCH-23390 (0.032 mg/kg, s.c.) and returned to the apparatus for 30 min. After 30 min mice were administered PF-1642 (10 mg/kg, s.c.) or vehicle control (s.c.) and returned to the apparatus for an additional 2 h during which time cumulative activity was recorded (**Figure 2C**). Data were compared within genotype using a two-way ANOVA with a Dunnett’s post-hoc test versus vehicle treated group.

#### Rat Electroencephalography and Polysomnography

Model 4ET telemetry device components (Data Sciences International, St Paul, MN, USA) were bilaterally placed in subcutaneous pockets on the dorsal flank of adult male Sprague-Dawley rats (200–400 grams). Two pairs of leads were implanted superficially to burr holes drilled over the frontal cortex (stainless steel screws, Plastics One, Roanoke, VA at coordinates: A-P = +1.5 mm, M-L = 1.5 mm) and parietal cortex (coordinates: A-P = −3.7 mm, M-L = −2.2 mm), and the cerebellum bilaterally to be used as ground and reference for electroencephalographic (EEG) recordings. A stainless-steel wire (Plastics One, Roanoke, VA) was implanted into the neck muscle to monitor electromyogram (EMG). All recordings were performed inside the home cages of animals using RPC-2 telemetry receivers (Data Sciences International, St. Paul, MN) at a sampling rate of 500 Hz for data acquisition. Baseline data, while on vehicle, were obtained for 24 h prior to compound administration. PF-6142 (1.0 or 5.6 mg/kg, s.c.) was administered acutely following the baseline day.

Raw EEG traces were analyzed using custom scripts in MATLAB (The Mathworks, Natick, MA, version 7.8 (R2009a)) to evaluate spectral changes between treatment groups. Raw EEG data were read into the MATLAB software, segmented to match that of the polysomnography (PSG) data (see below) and fast Fourier transforms (FFTs) were performed. Only data collected on the parietal lead were the subject of statistical analyses. PSG analysis was applied to all EEG/EMG data and utilized an in-house algorithm developed in LabView (National Instruments, Austin TX) as previously described (Harvey et al., 2013).

Relative power data in each band aggregated over 2 h-long time bins, while on drug, were normalized to baseline levels in corresponding time bins relative to dosing. For statistical analysis the R software was used. The effects of two within factors, dose and time, on cumulative time spent in three stages, awake, REM, and NREM sleep were assessed using generalized linear mixed model. The model specification explicitly accounted for a crossover design with repeated measures by introducing auxiliary factors, day, treatment sequence, and day by time product. To account for correlations within subjects, we employed a first order autoregressive scheme, which assumes that correlations decay exponentially with the lag between the measurements. The model was fitted using the method of restricted maximum likelihood. Significant findings were followed by least significant difference tests for pairwise differences across doses and across doses at fixed times for treatment and treatment by time factors, respectively. Tukey-Kramer procedure was used to adjust for multiple hypothesis testing. For all statistical tests, p < 0.05 was considered significant. The same fixed factors and type of statistical model were used to explain cumulative power within EEG power bands; the power was log transformed before the analysis.

#### Mouse Prepulse Inhibition (PPI)

Drug and behaviorally naïve adult male C57BL/6J mice (9–11 weeks of age; n=8 per dose group) were used for PPI experiments. Subjects were tested individually in SR-Lab acoustic startle chambers (San Diego Instruments, San Diego CA, USA) equipped with a restrainer mounted atop a piezoelectric accelerometer which measured transduced movement in response to the presentation of audio stimuli presented through a speaker mounted 20 cm above the animal. Subjects were acclimated to an anteroom adjacent to the testing room at minimum 60 min prior to testing. Test sessions began with a 5 min acclimation period to background noise (65 dB) followed by presentation of six randomized repetitions of the 120 dB startle stimulus (40 ms duration) presented alone or paired in combination with a pre-pulse stimulus of 68, 72, or 74 db (20 msec in duration) presented 80 msec prior to the 120 dB startle stimulus, which was equivalent to +3, +7, and +9 db over background noise, respectively. Data were also recorded for no stimulus values to evaluate background level of response. The inter-trial interval between stimulus presentations was randomized and ranged from 10 to 20 s. Test compounds were administered 30 min (s.c.) prior to testing. For experiments evaluating both PF-6142 and risperidone, each compound was administered at a different injection site (s.c.) with PF-6142 injected immediately prior to risperidone. Percent PPI was calculated for each individual subject as the relative change in the 120 dB startle response in the presence of each prepulse intensity using the formula: 100-((prepulse-pulse)/pulse)*100 as previously described (Ralph-Williams et al., 2003).

The experiment was analyzed using a two-way mixed model ANOVA (lme4 library in R software). The model included prepulse stimulus intensity levels, treatment (as all combinations of pre-treatment and treatment), and their interaction as fixed factors and random intercept, random slope for each animal as random factors. Significant ANOVA results were followed by planned post-hoc contrasts of least squared means across treatment arms at each prepulse stimulus intensity levels, slopes with respect to prepulse stimulus intensity levels, and planned contrast between slopes. Three-way ANOVA model with pre-treatment and treatment handled as independent factors yielded the same findings for all planned comparisons (not reported here). In order to adjust for multiple hypothesis testing we used false discover rate method which controls the expected proportion of false discoveries among the rejected hypotheses. For all statistical tests, p < 0.05 between groups was considered significant.

#### Rat Conditioned Avoidance Response (CAR)

The CAR assay was performed under similar conditions previously described (Marquis et al., 2011) at WuXiAppTec Inc. (Delin Rd. #90, Waigaoquiao Free Trade Zone, Shanghai 200131, China). For this study, adult male Fisher-344 rats were trained and tested in a two-way active avoidance apparatus with MED-PC software (MED Associates, St. Albans, VT, USA).

Briefly, subjects were handled and acclimated to the shuttle boxes for 2 d prior to training sessions. During training sessions, subjects were trained to avoid an electric footshock by moving to the adjacent, non-stimulus side of the shuttle box upon presentation of tone + light stimuli which preceded the presentation of the footshock (0.6 mA, 10 s duration) by 10 s. For this assay an avoidance was defined as moving to the adjacent compartment during the tone+light presentation that preceded the shock, an escape was defined as moving to the adjacent compartment upon presentation of the shock, and an escape failure was defined as a lack of relocation to the adjacent compartment throughout the presentation of the shock. Subjects received 30 trials of training per day for 5 d. Subjects with ≥ 80% avoidance responses on two consecutive days with no escape failures, were considered qualified for testing, and were randomized across treatment groups (n=8–9 per treatment group). PF-6142 (1.78 mg/kg) and risperidone (0.1 or 0.56 mg/kg), and their respective vehicles were administered (s.c.) 60 and 30 min, respectively, prior to the start of the experiment at separate injection sites. Avoidance responding was calculated as % of the number of total trials in which an avoidance occurred. Data were calculated for each animal and compared across treatment groups using one-way ANOVA with Dunnett’s post-test versus the vehicle + vehicle treated group.

#### Ketamine-Disrupted RAM Experiment

Two cohorts of adult male Long-Evans rats (N=30 each) were trained in a spatial working memory task on an eight-arm RAM, (Pathfinder Maze System, Lafayette Instrument Co., Lafayette, IN), using a procedure adapted from Ward et al. (1990) and described in detail in Strick et al. (2011). Briefly, rats were food-restricted to provide motivation to perform the RAM task. The task requires that the animals enter each arm to retrieve a reinforcement food pellets, using spatial cues in the room to remember which arms of the maze they have previously entered. Rats were individually placed on the maze and allowed to navigate until all eight arms were entered and the pellets were consumed or until 30 choices were made, or until 5 min had elapsed. Entry into an arm previously entered was counted as an error. If an animal failed to choose all eight arms in 5 min, the arms not chosen were also counted as errors. Training continued until all animals had reached the training criterion, defined as two or fewer errors on two consecutive days. Administration of the NMDA antagonist, ketamine, to well-trained rats consistently produces significant disruption of performance in the RAM task, resulting in a significant increase in the number of working memory errors. This study was designed to test the ability of D1 agonists to reverse ketamine-induced working memory deficits in well-trained rats. On test days, animals that met training criteria were randomly assigned to treatment groups and administered vehicle or PF-6142 (0.01, 0.056, 0.178, 0.56 mg/kg, s.c.), followed 90 min later by administration of ketamine (10 mg/kg, s.c.). Performance on the maze was evaluated 30 min later by an observer that was blinded to treatments.

The R 3.0.1 statistical software was used to compare the error rate data. The effects of treatment and the interaction on the error rate were assessed using a one-way mixed model ANOVA using generalized least squares method from lme4 library. Significant ANOVA results were followed by post-hoc pairwise comparisons of least squared means across treatment arms. In order to adjust for multiple hypothesis testing we used false discover rate method which controls the expected proportion of false discoveries among the rejected hypotheses. For all statistical tests, p < 0.05 between groups was considered significant.

#### SDR in the Nonhuman Primate

Administration of the NMDA antagonist ketamine consistently produces significant disruption of performance in the SDR task in nonhuman primates, resulting in a significant increase in the number of working memory errors. This study was designed to test the hypothesis that PF-6142 would provide significant protection versus the ketamine-induced working memory deficits in nonhuman primates.

##### Cognitive Testing

Rhesus monkeys were trained to stability on a variable SDR task in a sound-attenuated Wisconsin General Testing Apparatus (Roberts et al., 2010). Briefly, subjects observe while the investigator baiting one of two to seven wells with a highly preferred food reward and then covers all the wells with identical square plaques. An opaque screen is then lowered for one of five variable delays, which are pseudorandomized across trials within a session. Thus, delays are defined as 0–4 N, where “N” is a value that is animal dependent and ranges from 1 to 10 s depending upon the difficulty level of the task at which an animal reaches the criterion of stable performance. At the end of the delay period, the opaque screen is raised, and the animal must select the well that had been baited to obtain a reward. Each test session consists of 20 trials wherein both the baited well and delay length are pseudorandomized across trials. Before study initiation, all subjects were required to reach stability over a period of 10 consecutive test sessions, where stability was defined as an average of 65–75% correct. Stability was attained by varying the number of wells and the delay value for each animal. Subjects were originally trained on a two-well board with an N value of 1. The N value and the number of wells were gradually increased until the animal consistently scored within stability range. Once stability was attained for a given number of wells and N value, that combination was kept constant throughout the course of the study (Roberts et al., 2010). The range of stable performance for the 10 subjects was two to five wells and an N value of 1–7 s (median, four-well testing board, N = 5 s). Data were transformed using logarithmic function [log(x+5)] to improve normality and analyzed using ANOVA.

##### Drug Administration

Subjects received pretreatment with vehicle (sterile saline solution) or PF-6142 (0.0015, 0.015, 0.15 mg/kg, s.c.) 4 h before cognitive testing. They then received an intramuscular injection of either vehicle (sterile saline) or ketamine (0.7–1.7 mg/kg; Fort Dodge Co.) 0.25 h before cognitive testing. The dose of ketamine for each animal was predetermined such that all animals achieved a comparable magnitude of cognitive impairment (e.g., a score of less than ~50% correct) relative to their pretreatment baseline performance of ~70% correct. Thus, for the present study, one monkey received 0.7 mg/kg, seven monkeys received 1.0 mg/kg, and two monkeys received 1.7 mg/kg of ketamine. The vehicle/PF-6142 and vehicle/ketamine treatments were assigned using a randomized Latin square design with a total of eight conditions. Except for the vehicle/vehicle condition, there was a minimum 2-week washout period between all acute challenges during which time animals were required to “restabilize” to baseline performance levels, which was defined as at least three consecutive testing sessions wherein cognitive performance ranged between 65% and 75% correct.

#### PPF and Delta Field Potential Oscillation Power Measurement in Rat

Experiments were performed on n=10 adult male Sprague-Dawley rats (275–305 g) under urethane anesthesia (1.5 g/kg, i.p.). The femoral vein was cannulated for i.v. administration of drugs. A stimulation electrode was placed in the CA1/subiculum region (coordinates: A-P = +6.3 mm, M–L = +5.2 mm, D-V = +8.0 mm) using stereotactic methods and unilateral local field potential (LFP) was recorded by a metal monopolar macroelectrode placed into the medial PFC (mPFC; coordinates: A-P = +3.0 mm, M-L = +0.6 mm, D-V = +5.0 mm). The LFP was amplified, filtered (0.1–100 Hz), displayed and recorded for on-line and off-line analysis (Spike2 program, CED, Cambridge, UK). Evoked responses to the first and the second stimuli were identified (P1 and P2, respectively) and the amount of PPF determined by the formula: (P2 amplitude/P1 amplitude). Waveform averages used to calculate PPF consisted of 60 consecutive stimuli. Five minutes were allowed between administration of each drug dose and the starting of the subsequent average of each 10-min period after each cumulative dose (0.1–1 mg/kg, IV). Disruption in power of LFP delta activity was measured as the percentage of power in low frequency (0–1.8 Hz) irregular activity in the total (0–4 Hz) delta power range. LFP power spectra were determined during periods concurrent with waveform averages and PPF calculation. Statistical significance was determined by means of two-tailed paired Student’s t-test.

#### Plasma Protein Binding and PK Studies

Across the set of experiments, PK was either collected from satellite animals or in separate, dedicated studies. In addition to plasma PK, exposures were obtained in brain tissue for rat and mouse, and plasma protein binding and brain tissue binding were measured. Using the measured exposures, partitioning, and binding parameters, and the

$$RO\left(\%\right)=\;\frac{C_{\text{b;u}}\left(\text{nM}\right)}{C_{\text{b;u}}\left(\text{nM}\right)+K_{i}\left(\text{nM}\right)}\cdot 100$$

equation, a correlated receptor occupancy estimate (RO) was calculated for each of the exposures presented in **Table 2**.

**Table 2:** Representative Exposure Data.

Species	Dose (mg/kg)	Time (h)	PF-6142 plasma concentration (ng/ml)	Calculated brain D1R occupancy estimate (%)
mouse	5.6 (SC)	1	332	~25
rat	10 (SC)	1.5	1260	~55
NHP	0.1 (SC)	0.5	31	~20

## Results

### Acute and Sub-Chronic PF-6142 Increases ACh Levels in Rat and Mouse and the Effect Is Attenuated in the PFC of D1 Knockout Mouse

**Figure 1:**
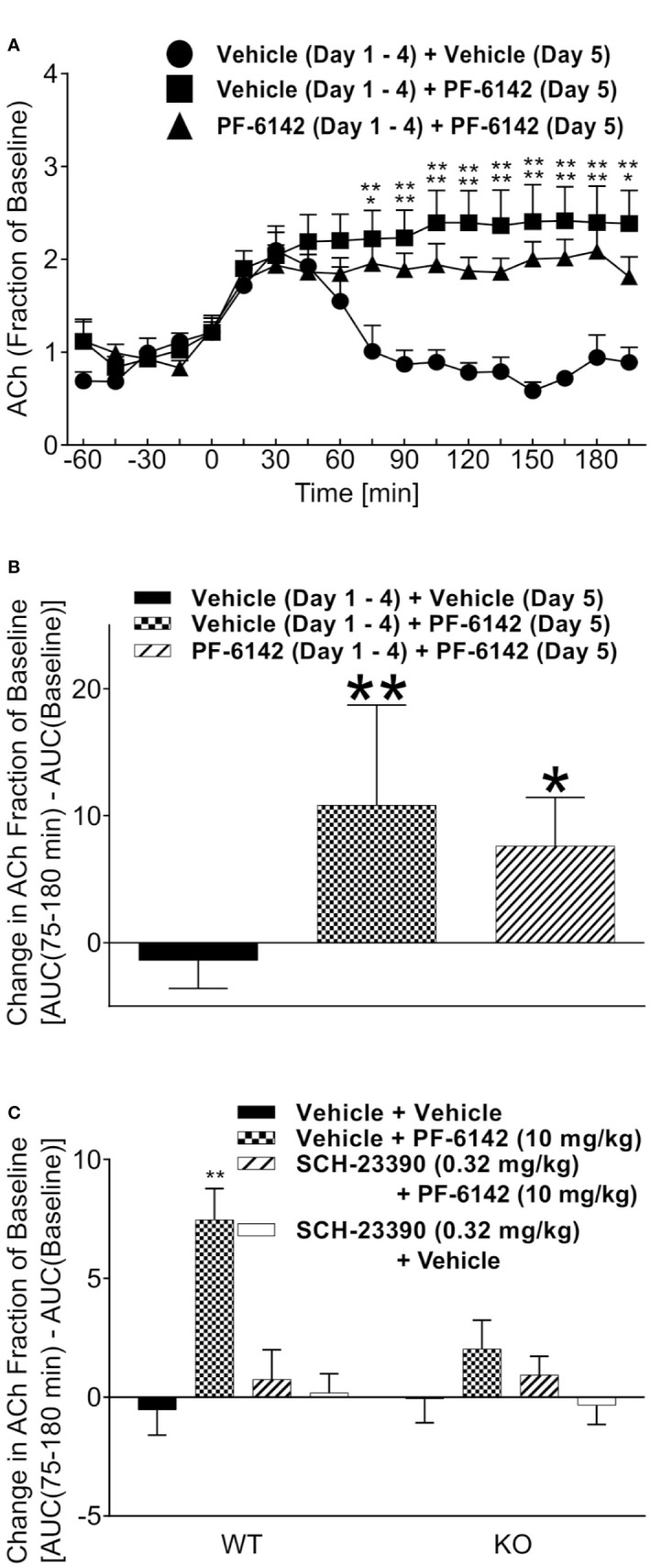
Acetylcholine following administration of PF-6142 in rat and mouse. Acetylcholine levels in the rat prefrontal cortex (PFC) following subchronic dosing with PF-6142. **(A)** Time course data comparing the effect of vehicle or PF-6142 (10 mg/kg, SC) on ACh levels in the rat PFC after repeated dosing for 5 d. **(B)** 75–180-min total area under the curve of time course data. Points represent the mean + SEM, One-way ANOVA with Dunnett’s post-test adjusted *p < 0.05, **p < 0.01 vs. vehicle. N = 7–8. **(C)** wild-type (WT) and D1 KO mouse acetylcholine levels in the PFC. 75–180-min total area under the curve (AUC) data for WT and D1 KO mice treated with PF-6142 (10 mg/kg, SC) and SCH-23390 (0.32 mg/kg, SC). Two-way ANOVA with Tukey’s post-test adjusted *p < 0.05, **p < 0.01 vs. vehicle + vehicle within genotype. N = 6–7.

Time course data showing the ability of PF-6142 (10 mg/kg, SC) to increase ACh levels in the rat PFC after five consecutive days of treatment is presented in **Figure 1A**. An analysis of the time course data indicates that treatment with vehicle followed by PF-6142 on day 5 [vehicle (sub-chronic) + PF-6142 (acute) group on **Figure 1A**] increased cortical ACh levels in dose dependent manner post-dose as compared to 5 d of vehicle treatment [vehicle (subchronic) + vehicle (acute) group seen in **Figure 1A**, *F*(treatment)_2, 19_ = 24.74, *p* = 0.0101, *F*(time)_17, 323_ = 17.76, *p* < 0.0001, *F*(interaction)_3, 42_ = 6.224, *p* = 0.023]. A comparison of the overall responses on day 5, expressed as the change in the area under the curve over the 75–180 min post-treatment time period, also shows that the PF-6142 mediated increase in cortical ACh was maintained after repeated dosing for five consecutive days (*F*_2, 19_ = 12.09, *p* = 0.0004; **Figure 1B**). Further, acute effects of PF-6142 and SCH-23390 in DR knockout and WT mice revealed an effect of treatment (*F*_2, 42_ = 9.183, *p* < 0.0001) and interaction (*F*_3, 42_ = 3.4, *p* = 0.0263; **Figure 1C**). Post-hoc analysis revealed that only the PF-6142 treatment in the WT group but not in the KO group had increased ACh levels when compared to vehicle.

### PF-6142 Acutely and Subchronically Increases Locomotor Activity in Mice and Is Attenuated by D1 Receptor Blockade

**Figure 2:**
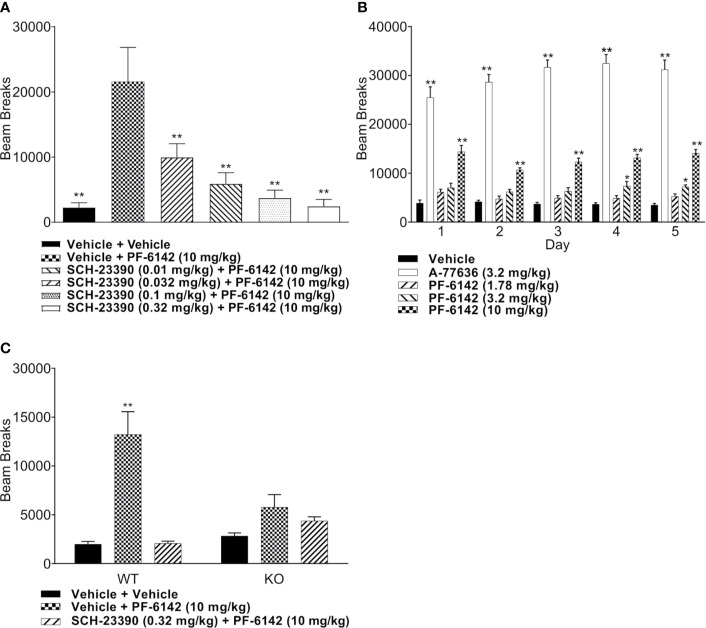
Mouse locomotor activity (LMA) following administration of PF-6142. **(A)** Treatment with PF-6142 increases the number of beam breaks and pretreatment with the D1 antagonist, SCH-23390, effectively and dose dependently blocks the hyperactivity induced by PF-6142. **(B)** Daily administration of D1 agonists. **(C)** The hyperactive response is greatly diminished in the D1 KO mice compared to WT mice. Data are shown as the mean beam breaks + SEM. N = 8. One-way ANOVA with Dunnett’s post-test adjusted *p < 0.05, **p < 0.01 vehicle + PF-6142 (10 mg/kg) **(A)**. Two-way ANOVA with Dunnett’s **(B)** or Tukey’s **(C)** post-test adjusted *p < 0.05, **p < 0.01 vs vehicle **(B)** or vehicle + vehicle **(C)**.

PF-6142 dose-dependently increased horizontal activity (*F*_4,35_ = 9.509, *p* < 0.0001) and achieved significance at the 10 mg/kg dose (*p* < 0.0001). To confirm D1 selectivity of PF-6142, animals were pretreated with D1 antagonist SCH-23390 (0.01, 0.032, 0.1, and 0.32 mg/kg, s.c.) in **Figure 2A** which showed a strong effect of treatment (*F*_4,42_ = 57.77, *p* < 0.0001). SCH-23390 blocked PF-6142 (10 mg/kg; s.c.)-stimulated activity in mice (*p*<0.0001). Locomotor activity data from five consecutive days of dosing with D1 agonists are presented in **Figure 2B**. A-77636 a selective D1 receptor full agonist (3.2 mg/kg, s.c.) and PF-6142 (10 mg/kg, s.c.) increased locomotor activity in CD-1 mice [*F*(treatment)_4, 175_ = 577.2, *p* < 0.0001, *F*(day)_4, 175_ = 1.817, *p* = 0.1276, *F*(interaction)_16, 175_ = 2.319, *p *= 0.0040]. PF-6142 (1.78 mg/kg) did not significantly increase horizontal activity while PF-6142 (3.2 mg/kg, s.c.) only increased activity significantly on days 4 and 5 of testing (*p* = 0.0311 and *p* = 0.0309, respectively). The daily comparison of the 3.2 mg/kg group alone did not reveal any changes between days 1–5 (*F*_4, 35_ = 0.5469, *p* = 0.7025). WT and D1 KO mice were treated with PF–6142 (10 mg/kg; s.c.) and data is presented in **Figure 2C** [*F*(treatment)_2, 42_ = 23.72, *p* < 0.0001 and *F*(interaction)_2, 42_ = 10.91, *p* = 0.002]. PF-6142 increased activity in the WT mice (*p* < 0.0001) and the effect was attenuated by SCH-23390. The KO mice did not show hyperactivity in response to PF-6142 treatment (*p* = 0.6731).

### *In Vivo* Freely Moving Electrophysiological Recordings

#### PF-6142 Significantly and Dose-Dependently Decreases Delta and Increases Beta and Gamma Oscillation Power

**Figure 3:**
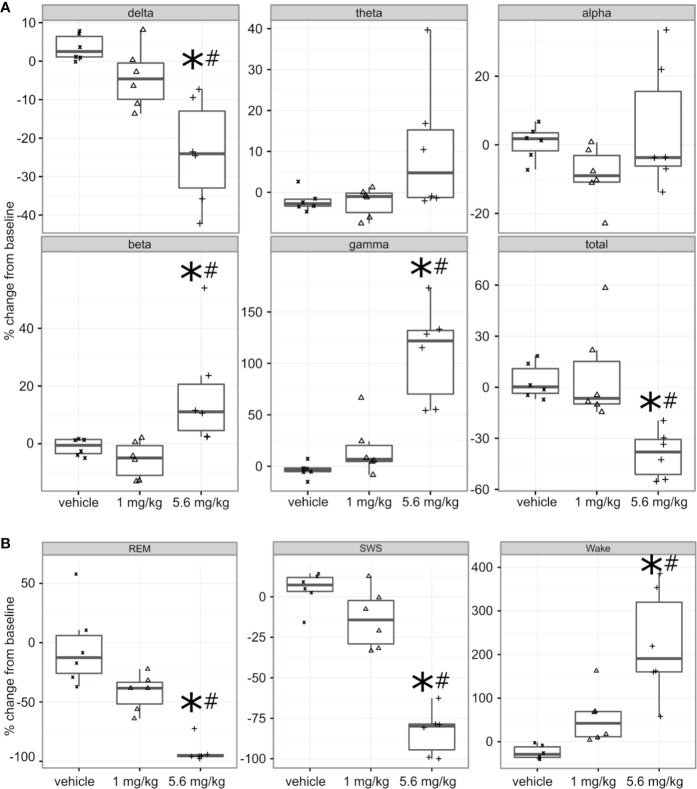
Quantitative electroencephalography and polysomnography analysis of treatment with PF-6142. **(A)** Systemic PF-6142 treatment significantly and dose dependently modulates oscillatory power in freely moving rats. Vehicle or PF-6142 (low: 1.0 mg/kg, high: 5.6 mg/kg, SC) was administered in the morning during the inactive period of the animals in their home cage. Delta (0.5–4 Hz), theta (4–9 Hz), alpha (9–13 Hz), beta (13–28 Hz), and gamma (28–80 Hz) oscillation power was analyzed during the first 4 h following treatment together with the total power contained in the signal. Statistical analyses revealed that treatment only with the high dose resulted in significant (*p < 0.05 vs. vehicle, #*p* < 0.05 vs. low) decrease of delta and total powers and increase of beta and gamma powers. Symbols (x for vehicle, Δ for low, and + for high dose) show values for individual animals. All animals are shown, outliers are not indicated separately, the upper and lower hinges on the boxplots show the 25^th^ and the 75^th^ percentiles, respectively, horizontal bar in the boxplot shows median value, whiskers extend to the minimum and the maximum values. **(B)** Systemic PF-6142 treatment significantly and dose dependently increases the time spent in wakefulness in freely moving rats. The fraction of time rats spent awake (Wake), in slow-wave sleep (SWS), or in REM sleep during the first 4 h following treatment were analyzed. Statistical analyses revealed that treatment with the high dose (5.6 mg/kg, SC) resulted in a significant (*p < 0.05 vs. vehicle, ^#^*p* < 0.05 vs. low) decrease of the time spent in SWS and REM sleep, and an increase of the time spent awake. Boxplots are set up as described in **(A)**.

Freely moving animals were dosed with PF-6142 in their home cage. Electroencephalographic (EEG) data were recorded for 24 h following treatment to allow for the monitoring of long-term effects (**Figure 3A**). To remain within the expected window of treatment-related effects, time-collapsed statistical analyses of the quantitative EEG data were limited to the first 4 h. The statistical model revealed that during the first 4 h following treatment PF-6142 significantly decreased the change in delta oscillation power from its baseline value (*F*_2, 10_ = 12.8, *p* = 0.002). Post-hoc testing showed that the high dose (5.6 mg/kg, s.c.; *t*_15_ = 5.0, *p* = 0.0004) but not the low dose (1.0 mg/kg, s.c.; p = 0.3) resulted in significant delta power decrease relative to the vehicle treatment. Contrary to the changes in delta power, PF-6142 treatment resulted in a significant increase in beta (*F*_2, 10_ = 5.9, *p* = 0.02) and gamma (*F*_2, 10_ = 21.3, *p* = 0.0003) powers. Post-hoc tests again showed that only the high dose resulted in a significant change (*t*_15_ = −2.7, *p* = 0.04 for beta, and *t*_15_ = −6.3, *p* < 0.0001 for gamma). Power in other studied frequency bands was not found to change significantly, however the total power in the EEG signal decreased significantly (*F*_2, 10_ = 10.3, *p* = 0.004) with post-hoc testing confirming that only treatment with the high dose created a significant total power decrease (*t*_15_ = 3.9, *p* = 0.004).

#### PF-6142 Significantly and Dose Dependently Decreases the Time Spent in Sleep and Increases the Time Spent Awake

To further elucidate the effects of a D1 agonist on the vigilance state of freely moving rats housed and studied in their home cages, EEG and EMG data was subjected to PSG analysis (**Figure 3B**). Like the quantitative EEG analysis, the time-collapsed statistical analyses were limited to only the first 4 h following treatment. The statistical model revealed that treatment effects were significant for changes in the fraction of time spent in rapid eye movement (REM) sleep, (*F*_2,8_ = 32.7, *p* = 0.0001). Post-hoc pairwise comparisons *via* least squares means identified that the vehicle group had 88.2% higher fraction of REM sleep compared to the high dose group, which was significant (*t*_11 _= 6.15, *p* = 0.0002). Similarly, time spent in slow-wave sleep (SWS) was also found to significantly decrease (*F*_2, 10_ = 56.8, *p* < 0.0001), again with an 88.1% decrease relative to the vehicle group achieved following the administration of the high dose (*t*_15_ = 10.3, *p* < 0.0001). Since the D1 agonist suppressed sleep, it was expected that animals would spend more time awake. This was supported by the statistical analysis which confirmed that there was a significant increase of the time spent in wakefulness (*F*_2, 9_ = 14.3, *p* = 0.001). Animals spent significantly more time awake following the high dose (246.9% increase, *t*_15_ = −5.3, *p* = 0.0003).

### PF-6142 Does Not Impact the Effect of Risperidone on Mouse Startle and PPI

**Figure 4:**
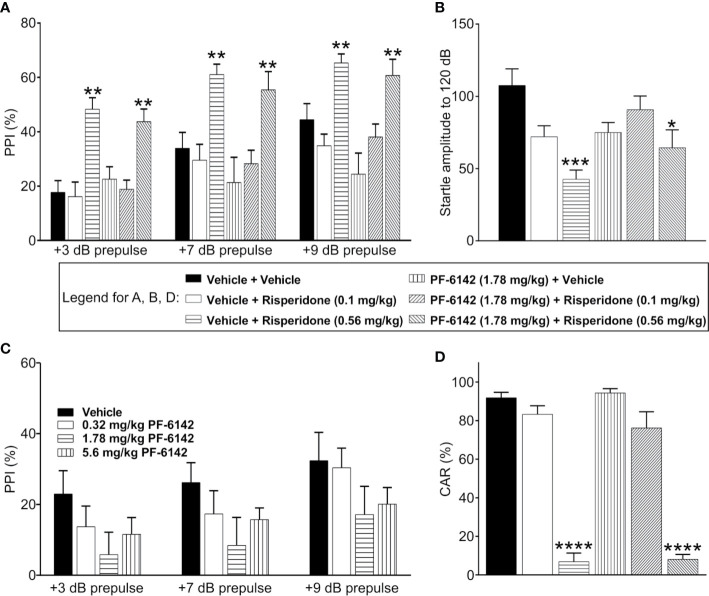
PF-6142 Does not alter the antipsychotic-like activity of risperidone in rodents. **(A)** As expected, risperidone produced increases % PPI in adult male C57BL/6J mice consistent with an antipsychotic-like profile in this assay. Administration of PF-6142 alone or in combination with risperidone does not alter PPI responses (n=8 per treatment group). **(B)** Statistically significant reductions in startle responses were observed with the highest dose of risperidone (0.56 mg/kg) which was not altered in the presence of PF-6142. (**C)** PF-6142 alone produced modest non-significant reductions in % PPI in adult C57BL/6J mice. (**D)** Risperidone produces significant reductions in avoidance responding consistent with antipsychotic-like activity in adult male rats (n=8–9 per treatment) in the conditioned avoidance responding assay. There is no effect of PF-6142 alone or in combination with risperidone in avoidance responding. One-way ANOVA with Tukey’s post-test adjusted *p < 0.05, **p < 0.01 vs. vehicle + Risperidone (0.56 mg/kg) ***p < 0.001 vs. vehicle + vehicle. Note:2-way RM ANOVA (A). One-way ANOVA with Dunnett’s post-hoc test ****p < 0.0001 vs. vehicle + vehicle **(D)**.

Consistent with previous published reports, vehicle treated C57BL/6J mice demonstrated prepulse dependent increases in % PPI which was dose dependently increased by pretreatment with risperidone (0.1–0.56 mg/kg) (**Figure 4A**). ANOVA revealed that the effect of prepulse stimulus intensity level was significant [*F*(dB)_1, 42_ = 95.9, *p* < 2.1·10^−12^], the effect of treatment was significant [*F*(treatment) _5, 42_ = 8.67, *p* = 1.04·10^−5^], and their interaction was significant [*F*(int)_5, 42_ = 3.94, *p* = 0.005]. Post-hoc analysis showed that risperidone dose-dependently increased PPI at all pre-pulse intensities as expected, resulting in significantly higher PPI values following high dose risperidone treatment than vehicle for all decibel levels (*t*_42_ = 4.79, *p* = 0.0001 for 3 dB; *t*_42_ = 3.4, *p* = 0.004 for 7 dB; *t*_42_ = 2.63, *p* = 0.029 for 9 dB). However, consistent with the known side effect profile of risperidone, acoustic startle responses (120 dB) were dose dependently and significantly reduced (**Figure 4B**). Therefore, for the evaluation of PF-6142, experiments were conducted both with an ineffective dose of risperidone that did not alter startle responses (0.1 mg/kg) and a high dose of risperidone (0.56 mg/kg). For these combination experiments, to assess whether D1 agonism affected PPI, PF-6142 at a dose of 1.78 mg/kg was co-administered with risperidone (**Figure 4A**). This dose was selected as the highest dose of PF-6142 that in pilot experiments (**Figure 4C**) produced a modest but not significant reduction in %PPI. As expected, risperidone at only the high dose (0.56 mg/kg) but not the low dose (0.1 mg/kg) produced an increase in %PPI. In combination with risperidone, there was no effect of PF-6142 (1.78 mg/kg) on %PPI across prepulse intensities (*t*_42_ = −0.74, *p* = 0.477 for 3 dB; *t*_42_ = −0.67, *p* = 0.5044 for 7 dB; *t*_42_ = −0.59, *p* = 0.5555 for 9 dB). PF-6142 (1.78 mg/kg) produced modest impairments in %PPI which was significant relative to vehicle treated control at only the 9 dB prepulse intensity (*t*_42_ = −2.43, *p* = 0.029) which was not unexpected based modest reductions in %PPI observed in previous data (**Figure 4C**). Importantly the presence of PF-6142 did not alter risperidone’s effects on PPI.

### PF-6142 Has No Effect on CAR Alone or in the Presence of Risperidone in Rats.

PF-6142 was tested in the rat CAR assay for antipsychotic-like activity alone and in the presence of risperidone. PF-6142 alone (0.32–5.6 mg/kg, s.c.). did not alter % avoidance responses which relative to vehicle treated control. Mean % avoidance responses for PF-6142 were 95.93, 98.47, 95.57, and 95.75% for PF-6142 at doses of 0.32, 1.78, 3.2, and 5.6 mg/kg, respectively, were analyzed with one-way ANOVA versus vehicle treated controls (*F*_4, 37_ = 0.6378, *p* = 0.64). Therefore, for combination studies, of PF-6142 with risperidone, a dose of 1.78 mg/kg PF-6142 was selected. As presented in **Figure 4D**, risperidone (0.1–0.56 mg/kg, s.c.) produced the expected dose-dependent reductions in avoidance responding consistent with an antipsychotic-like profile in this assay. Mean % avoidance responses for vehicle, 0.1, and 0.56 mg/kg risperidone were 91.88, 83.33, and 6.875%, respectively with significant reductions at 0.56 mg/kg risperidone (*p* < 0.001). PF-6142 (1.78 mg/kg, s.c.) resulted in % avoidance responding of 94.38% which was not different than vehicle. In the presence of risperidone at either dose, PF-6142 did not alter % avoidance responses produced by risperidone which were 76.25 and 8.125%, respectively. One-way ANOVA versus vehicle treated controls revealed an effect of treatment with significant reductions observed only with 0.56 mg/kg risperidone alone or with 0.56 mg/kg risperidone in combination with PF-6142 (*F*_5, 43_ = 78.75, *p* < 0.0001).

### PF-6142 Reversed Ketamine-Induced Deficits in RAM Performance in Rats

**Figure 5:**
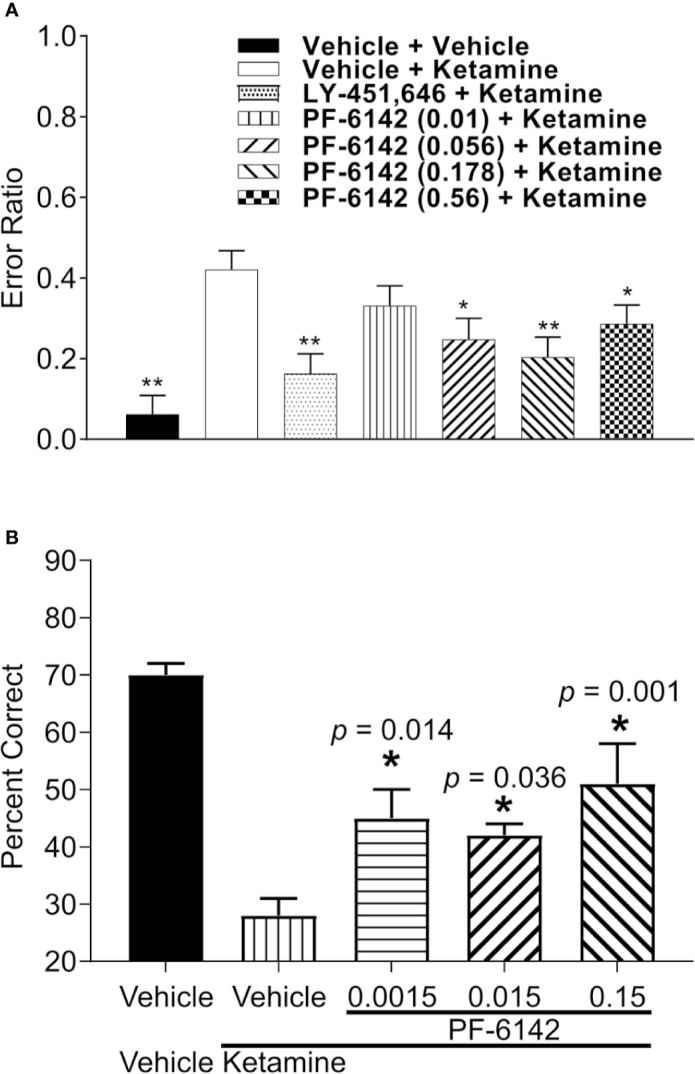
PF-6142 effects on ketamine-induced working memory deficits. **(A)** Pre-treatment with PF-6142 dose-dependently prevents ketamine-induced deficits in the rat radial arm maze assay. Data are presented as mean errors + SEM. Kruskal-Wallis test with Dunn’s multiple comparison post-test *p < 0.05, **p < 0.01 vs. vehicle + ketamine. N=10–21. **(B)** Treatment with PF-6142 prevents ketamine-induced deficits in the non-human primate spatial delayed response task. One-way ANOVA with Dunnett’s post-test adjusted *p < 0.05 vs. vehicle + Ketamine and N = 8 NHP.

Error ratio data from the rat RAM task are presented in **Figure 5A**. Ketamine (10 mg/kg, s.c.) treatment caused a robust increase in error rate (errors/choices) in rats trained to perform the RAM task. LY-451646 (0.32 mg/kg, s.c.), an α-amino-3-hydroxy-5-methyl-4-isoxazolepropionic acid (AMPA) receptor positive allosteric modulator, was used as a positive control and was shown to decrease the ketamine-induced error rate as expected. PF-6142 (0.01–0.56 mg/kg, s.c.) decreased the ketamine-induced error rate at all, except for the lowest dose administered (*F*_6, 48_ = 5.72, *p* = 0.0002).

### PF-6142 Ameliorates Ketamine-Induced Deficits in Nonhuman Primate Spatial Working Memory

Performance data (percent correct responses) from the NHP SDR task are presented in **Figure 5B**. Treatment with ketamine caused a robust decrease in percent correct on the SDR task from 70.63 ± 1.75% to 28.75 ± 2.45% [*F*(treatment)_1, 6_ = 62.089; *p* < 0.001]. Pretreatment with PF-6142 (0.0015–0.15 mg/kg, s.c.) significantly attenuated the ketamine-induced deficits in the task at all doses tested [*F*(pretreatment)_3, 18_ = 5.733; *p* = 0.006], improving performance by more than 10% correct (range 42.5–52.1%). Pretreatment followed by placebo (sterile saline) instead of ketamine indicated that PF-6142 had no effect on its own on working memory performance under normal conditions.

### *In Vivo* Anesthetized Electrophysiological Recordings

#### PF-6142 Significantly Reverses N-Methyl-D-aspartate (NMDA) Receptor Blockade-Induced Changes in Paired Pulse Facilitation (PPF)

**Figure 6:**
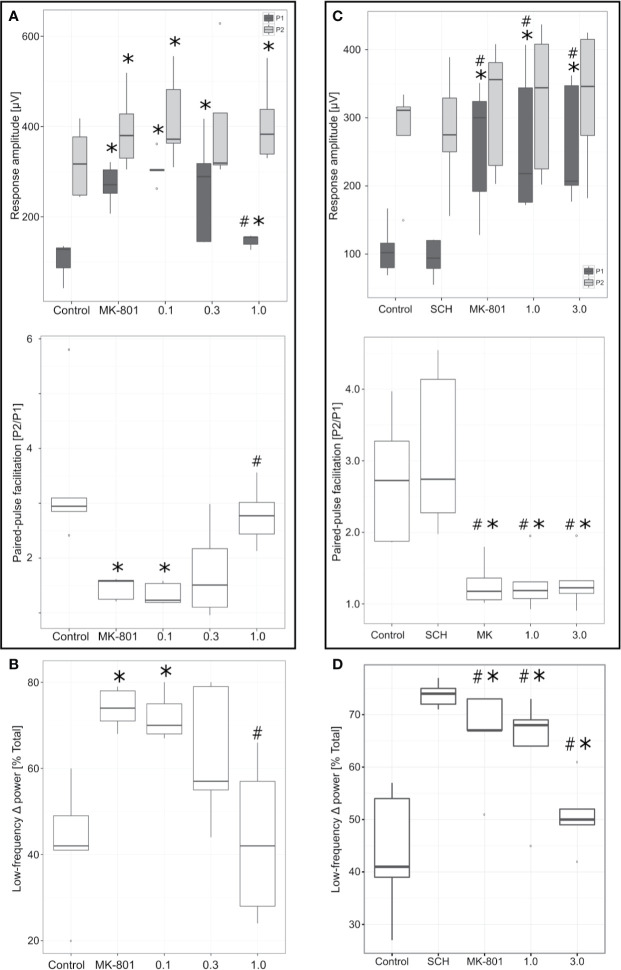
Effects of PF-6142 on NMDA antagonist disrupted paired-pulse facilitation (PPF) and delta oscillations. **(A)** PF-6142 significantly reverses NMDA blockade-induced changes in PPF. PPF (calculated as P2/P1) values as a function of drug treatment. Note that PF-6142 reverses MK-801-evoked decrease of PPF with an ED_50_ of 0.35 mg/kg (*: *p*<0.05 vs. Control, #: *p*<0.001 vs. MK-801; *n*=5). The upper and lower hinges on the boxplots show the 25^th^ and the 75^th^ percentiles, respectively, horizontal bar in the boxplot shows median value, whiskers extend to the minimum and the maximum values, “o” indicates data points outside of the 1.5*inter-quartile range of the hinges. **(B)** PF-6142 significantly reverses NMDA blockade-induced mPFC low frequency delta activity increase. Power contained in the low frequency delta (0–1.8 Hz) band expressed as a percentage of the total delta (0–4 Hz) power. Note that PF-6142 completely reverses MK-801 induced increase of low frequency delta oscillation (*: *p*<0.02 vs. Control; #: *p*<0.02 vs. MK-801; *n*=5). **(C)** Antagonism of D1Rs blocks effects of PF-6142 on PPF. Figure shows PPF values as a function of drug treatment. Note that administration of SCH-23390 alone had no effect on either the P1 or P2 components or PPF, while this pretreatment completely blocked PF-6142 effects even at high doses. **(D)** Antagonism of D1Rs blocks effects of PF-6142 on mPFC low frequency delta activity. Power contained in the low frequency delta (0–1.8 Hz) band expressed as a percentage of the total delta (0–4 Hz) power. Note that as with PPF SCH-23390 completely blocks effects of PF-6142 on reversing MK-801-induced changes (*p<0.05 vs. Control, ^#^*p*<0.03 vs. SCH-23390; *n*=5).

In agreement with previous studies (Kiss et al., 2011), administration of NMDA receptor antagonist MK-801 (0.1 mg/kg, IV) resulted in significant increase of both the P1 (*t*_4_ = −10.49, *p* = 0.0005) and P2 (*t*_4_ = −8.05, *p* = 0.001) response amplitudes (**Figure 6A**). However, magnitude of the increase of P1 amplitude was proportionately much greater than that of P2 (44% vs. 10%, respectively). The resulting effect was a significant decrease of the corresponding PPF (PPF = P1 amplitude/P2 amplitude; *t*_4_ = 2.99, *p* = 0.04). MK-801 (0.1 mg/kg, IV) decreased PPF and subsequent cumulative PF-6142 administration (0.1–1 mg/kg, IV) rescued this effect. The reversal of MK-801 was an all-or-none effect. Once the effective dose of PF-6142 was reached, the onset of reversal was rapid and almost maximal for each animal with minimal further effect observed after additional cumulative dosing. In two out of the five animals in this study a reversal effect was observed after a cumulative IV dose of 0.3 mg/kg of PF-6142 while the remaining three animals required the maximal cumulative dose of 1.0 mg/kg tested to reverse MK-801. On the population level, animals exhibited significantly higher PPF values following the administration of PF-6142 (1.0 mg/kg, IV) than under MK-801 challenge (*t*_4_ = −5.28, *p* = 0.006). The ED50 for PF-6142 reversal of MK-801-induced effects on PPF was 0.35 mg/kg (95% confidence levels = 0.21–0.57 mg/kg) as calculated using the Spearman-Karber method.

#### PF-6142 Significantly Reverses NMDA Receptor Blockade-Induced Medial PFC (mPFC) Low Frequency Delta Activity Increase

The effects of MK-801 and subsequent cumulative IV dosing of PF-6142 on mPFC low frequency delta activity are shown in **Figure 6B**. MK-801 (0.1 mg/kg, IV) resulted in a significant increase (*t*_4_ = −4.36, *p* = 0.01) in low frequency (0–1.8 Hz) irregular delta activity. Subsequent cumulative dosing of PF-6142 (0.1–1 mg/kg, IV) dose-dependently reversed MK-801-induced increases in low frequency delta that paralleled its reversal of MK-801-induced changes in PPF. At the highest dose animals showed significantly decreased low frequency power compared to MK-801 challenge (*t*_4_ = 4.49, *p* = 0.01).

#### Antagonism of D1Rs Blocks Effects of PF-6142 Both on PPF and mPFC Low Frequency Delta Activity

To test selectivity and specificity of PF-6142 in generating PPF and low delta activity effects, SCH-23390 was used to pretreat animals before MK-801 and subsequent PF-6142 administration (**Figures 6C, D**).

SCH-23390 (0.32 mg/kg, IV) had no effect on baseline P1 (*t*_4_ = 1. 47, *p* = 0.21) and P2 (*t*_4_ = −0.17, *p* = 0.86) response amplitudes (**Figure 6C**). Furthermore, subsequent administration of MK-801 (0.1 mg/kg, IV) still resulted in significant and selective increase in P1 response amplitude (*t*_4_ = −4.90, *p* = 0.008) in all five of the animals in this study causing significant decrease of PPF (*t*_4_ = 4.88, *p* = 0.008) similarly to previous results without D1R antagonism. SCH-23390 (0.32 mg/kg, IV), had no impact on control PPF (*t*_4_ = −2.53, *p* = 0.06), and did not prevent MK-801 from decreasing PPF (two-sample t-test assuming equal variances: *t*_8_ = 1.00, *p* = 0.34). SCH-23390 did, however, block the potential of PF-6142 (1–3 mg/kg, IV) to reverse the MK-801 effects: in four out of five animals cumulative IV administration of PF-6142 (1.0–3.0 mg/kg) had no effect on MK-801-induced changes in PPF, and therefore no significant effect of PF-6142 was detected (at the highest dose of 3 mg/kg, IV *t*_4_ = −0.14, *p* = 0.89). These effects were largely due to the effect on P1 while P2 was unaffected.

The effects of SCH-23390, MK-801, and subsequent cumulative IV dosing of PF-6142 on mPFC low frequency delta activity are shown in **Figure 6D**. SCH-23390 (0.32 mg/kg, IV) alone had no significant effect on mPFC low frequency delta activity (*t*_4_ = −1.53, *p* = 0.19). Subsequent MK-801 (0.1 mg/kg) administration resulted in a significant increase (*t*_4_ = −6.15, *p* = 0.003) in low frequency irregular delta activity, statistically similar to the case when treatment with SCH-23390 did not precede MK-801 treatment (two-sample t-test assuming equal variances: *t*_8_ = 0.09, *p* = 0.93). Similar to PPF, cumulative IV dosing of PF-6142 in animals pretreated with SCH-23390 had no effect on MK-801-induced increases in low frequency delta activity (at the highest dose of 3 mg/kg, IV *t*_4_ = 2.08, *p* = 0.11).

### Receptor Occupancy Estimate for PF-6142

Maximal exposure was observed to occur between 0.5 and 2 h following dosing. Exposures increased in a generally dose proportional manner across most of the doses used in the pharmacology studies, and inter animal variability was typically low. Exposures were obtained in brain tissues for rat and mouse, and plasma protein binding and brain tissue binding were measured. From these data (not presented), we observe that PF-6142 is fully brain penetrant, with unbound concentrations approaching unity between the brain and plasma compartments.

A selection of representative exposure data is presented in **Table 2** to help contextualize the results of these *in-vivo* pharmacology studies with PF-6142 with published data reported on other D1R agonists. To account for any species differences in the affinity of PF-6142 for binding to D1R in each of the test species, radiolabel displacement assays were conducted in tissue from each one (data not shown) and measured values used in the receptor occupancy calculation.

## Discussion

Prefrontal cortical (PFC) functional alterations have been associated with the symptoms of multiple neuropsychiatric and neurodegenerative diseases, such as schizophrenia and Parkinson’s disease (Howes and Kapur, 2009). In the PFC, DA D1Rs play a key role in cognitive control circuits that support working memory and executive function; thus, potentiation of these receptors offers a potential therapeutic pathway to counteract cognitive symptoms. Accordingly, a number of selective D1R agonists have been explored to date, all of which contain the catecholamine structural motif of DA itself, however, the catechol structural element imparts generally unfavorable aspects to their *in vivo* PKs (Zhang et al., 2009).

Recently, a novel chemotype of D1R agonists was described (Davoren et al., 2018; Gray et al., 2018; Soutschek et al., 2020b) that does not contain a catechol group and has generally good PKs and brain penetration. These compounds are reported to bind to the orthosteric DA site on D1Rs and activate cyclic adenosine monophosphate (cAMP). They also show evidence of biased G protein–coupled receptor (GPCR) signaling with respect to β-arrestin, with functional consequences on receptor internalization *in vitro* and on repeat-dose *in vivo* behavioral pharmacology. Recently, direct iontophoretic application of another compound from this new chemical series to aged monkeys performing a delay-dependent spatial working memory task yielded electrophysiologic evidence of D1R mediated excitatory actions on dlPFC task-related firing (Wang et al., 2019).

In this paper, a prototypical member of the novel non-catechol D1R agonist series, PF-6142, is characterized in various preclinical models. A specific goal of these studies was to assess if prior observations of selective D1R agonist pharmacology from *in vivo* models using catechol-based compounds (Roberts et al., 2010) would translate to these new compounds given their novel structure, signaling properties, high D1/5 selectivity, and different *in vivo* PKs. Assays were selected to cover different aspects of D1R-relevant circuitry with a general focus on cognitive and motor systems.

PF-6142 has moderate affinity for the human D1 and D5 receptors. Current literature is ambiguous regarding the differential expression, functional impact, and developmental changes of these receptor subtypes (Ciliax et al., 2000), thus effects observed in this paper can be attributed to an action *via* both D1 and/or D5 receptors. However, PFC dependent activity is likely due to the activation of D1 receptors that have higher cortical density including in PFC pyramidal cells in rats (Araki et al., 2007) and in NHP (Smiley et al., 1994; Montague et al., 2001; Lidow et al., 2003). The predominance of D1 was experimentally observed in experiments with D1R knockout mice where the effects of PF-6142 was absence.

D1R agonist-like activity was demonstrated by measuring aCh levels in the PFC of rats and mice using microdialysis. Like other D1R agonists, PF-6142 caused a robust increase in ACh level in the PFC which could be attenuated by administration of SCH-23390, a highly selective D1R antagonist in mice or by D1R knockout. Importantly, unlike currently available D1 agonists (Damsma et al., 1990; Imperato et al., 1994), the ACh release-promoting effect of PF-6142 was maintained following subchronic administration supporting previous observations that compounds from this non-catechol chemotype produce lasting functional effects without the rapid tolerance (Gray et al., 2018) that has been observed with catechol based D1 agonists (Kebabian et al., 1992).

Acute treatment of freely moving rats with PF-6142 resulted in a significant and dose-dependent increase of wakefulness and associated low-amplitude, high frequency electroencephalographic brain oscillations, primarily in the beta and gamma range. This stimulant activity is in line with increased selective activation of D1Rs (Herrera Solís et al., 2016) and is compatible with previous observations showing wake-promoting and EEG desynchronizing action of D1R agonist in normal (Ongini et al., 1985) and in a narcoleptic rodent model. Interestingly, it has also been shown recently that the D1R agonist SKF-38393 successfully alleviated excessive daytime sleepiness and restored REM sleep to baseline values in a macaque monkey model of Parkinson’s disease (Hyacinthe et al., 2014).

Similarly, to other D1R agonists, PF-6142 significantly and dose dependently increased locomotor activity in mice (Dracheva et al., 1999). Specificity of the response to D1R agonism was validated pharmacologically by administering the D1R antagonist SCH-23390 and by using a D1R knock-out mouse model. In both cases the hyperlocomotor response induced by PF-6142 administration was significantly attenuated. Importantly, the lack of effect in knockout mice suggests a D1R subtype-dependent action, although it does not fully discard the potential role of D5 receptors. Testing the effect of PF-6142 in D5R knockout mice is required to better understand the contribution of each receptor subtype.

Importantly, PF-6142 does not interfere or compromise the efficacy of risperidone, an antipsychotic drug, in the PPI assay and the CAR assay, two preclinical models used to demonstrate antipsychotic efficacy. All clinical antipsychotics agents show efficacy in these two preclinical models. Therefore, it is central to discard any potential interference with the standard care. These null results support the notion that D1R agonist administration will not interfere with the positive symptom efficacy of current antipsychotics medication that is likely used by patients.

Accumulating data suggesting that D1 receptors play a critical role in orchestrating function within the PFC and striatum for neuroadaptive processes which influence higher level functioning. Neuroimaging studies have shown increased D1R expression in PFC early in the course of illness in drug naïve schizophrenic patients and increased [^11^C]NNC 112 binding in the DLPFC was predictive of poor performance on a working memory task (Abi-Dargham et al., 2002).

This data has led to the premise that D1 receptor agonist therapy may ameliorate working memory impairment by modulating the insufficient DA tone in patients with schizophrenia.

To assess the potential of PF-6142 to improve working memory, two preclinical deficit models were used taking advantage of NMDA antagonism for inducing cognitive impairment. NMDA receptor (NMDAR) dysfunction can directly impact synaptic plasticity and modify circuit output. Previous studies have shown (Kiss et al., 2011) that systemic administration of the non-competitive NMDAR antagonist MK-801 disrupts short-term synaptic plasticity between hippocampal CA1 and the PFC and increases low frequency electrical activity in the PFC of anesthetized rats. Importantly, our results demonstrated that administration of PF-6142 significantly reversed these effects similarly to LY451395, an AMPAkine shown to reverse NMDA-antagonist-induced deficits in preclinical models of cognition in NHP (Roberts et al., 2010).

At the functional level, in the RAM assay, a rodent spatial working memory task, the partial D1R agonist PF-6142 reversed ketamine-induced deficits, another way to compromise NMDAR functioning, in a dose dependent manner.

Similarly, pretreatment with PF-6142 prior to an acute ketamine challenge prevented ketamine-induced impairment in the SDR task model of primate spatial working memory. At the doses PF-6142 was tested in both of these paradigms, PF-6142 appears not to show the expected U-type dose response pattern that was found previously for the partial agonist SKF38393 in this model, in contrast to the inverted-U-type response found for the full agonists SKF-81297 and A77636 (Zahrt et al., 1997; Roberts et al., 2010). However, assessment of wider dose range is required to confirm this observation.

The results of these studies highlight significant differences from previous observations and suggest wider efficacy window underscoring the therapeutic potential of this novel class of D1 agonist. However, it is reasonable to conclude the optimal dose of a D1R agonists for improving cognitive function in a disease state may vary according to individual differences and neuropsychiatric conditions and also suggest that dopaminergic treatments of psychiatric disorders should consider baseline DA levels in order to avoid side effects of over-or underdosing on cognition (Floresco, 2013).

These findings indicate this novel class of D1R agonists shows efficacy for improving functionality under conditions in which NMDAR transmission is impaired, as hypothesized in schizophrenia. We note the low doses of PF-6142 that were associated with reversal of ketamine-induced working memory deficit in rat and NHP, a finding consistent with data obtained using catechol D1 agonists. Independent of chemical class, the positive effects in these models occurred at low doses and consequently a very low estimated receptor occupancy (<5%; see **Table 2**). Given prior data on D1R full agonists in this model, additional study is warranted to fully understand the exposure-response relationship.

In summary, the collected data support the hypothesis that PF-6142 a novel, non-catechol-based compound has functional pharmacology that is generally consistent with the expected profile for a D1R agonist acting *via* increased cAMP signaling. The new tool has favorable PK properties compared to previously available D1R agonists that might enable further research on the D1R system, particularly chronic studies or paradigms which look to assess the impact of continuous D1R receptor occupancy over a sustained period. Taken together, results of these studies replicate published pharmacology and also extend what is known about the performance of D1R agonists in other models and provide data that encourages further development of D1R agonists as potential therapies for cognitive impairment in schizophrenia and other psychiatric illness.

## Dedications

The authors would like to dedicate this work to one of our co-authors, DJ, who passed away unexpectedly during the final stages of writing this manuscript. He was an extraordinary colleague whose commitment and expertise in drug discovery was critical to this work. DJ dedicated his life’s work to making a difference for patients, and he will be missed greatly.

## Data Availability Statement

The datasets presented in this article are not readily available because they contain proprietary information owned by Pfizer Inc. Requests to access the datasets should be directed to DG (david.gray@cerevel.com).

## Ethics Statement

The animal study was reviewed and approved by the Institutional Animal Care and Use Committee at Pfizer Inc. and Yale Animal Care and Use Committee.

## Author Contributions

RK, DG, TK, DJ, PS, SS, MH, GW, and SC contributed conception and design of the study. BH, WH, PS, RG, KK, KD, SS, JD, and AA conducted experiments. BH, WH, TK, DV, and KD performed data analysis. TK, RK, KD, DG, SS, MH, and JD wrote or significantly contributed to the writing of the manuscript. All authors contributed to the article and approved the submitted version.

## Funding

The authors declare that this study received funding from Pfizer Inc. The funder was not involved in the study design, collection, analysis, interpretation of data, the writing of this article or the decision to submit it for publication.

## Conflict of Interest

Authors were employees or collaborators who received funding from Pfizer Inc. SC and GW are recent consultants for UNITY Biotechnology Inc.
